# Incidental Appendiceal Adenocarcinoma Discovered After Ileocecectomy for Recurrent Small Bowel Obstruction: A Case Report

**DOI:** 10.7759/cureus.115908

**Published:** 2026-09-07

**Authors:** Aron Bhachawat, Heenal Marfatia, Suneetha Chintalapati, Kelly Winter

**Affiliations:** 1 Radiology, Obstetrics, and Gynecology, Kansas City University of Medicine and Biosciences, Kansas City, USA; 2 Family Medicine, Kansas City University of Medicine and Biosciences, Kansas City, USA; 3 Pathology, Ascension Via Christi, Wichita, USA; 4 Colorectal Surgery, Ascension Via Christi, Wichita, USA

**Keywords:** appendiceal pathology, ileocecectomy, metastatic recurrence, primary appendiceal adenocarcinoma, rectal adenocarcinoma, recurrent small bowel obstruction

## Abstract

We report a case of moderately differentiated appendiceal adenocarcinoma discovered on histopathologic examination following ileocecectomy for recurrent SBO in a woman in her early 50s with a history of stage III rectal adenocarcinoma in remission. Notably, serial surveillance imaging over two years had demonstrated a radiographically normal appendix prior to the obstructive presentation. Immunohistochemical and histopathologic features confirmed a metachronous primary malignancy rather than metastatic recurrence. This case exemplifies the diagnostic challenge posed by occult appendiceal neoplasms, the importance of meticulous histopathologic evaluation of all resected specimens, and the need to consider rare malignant etiologies in patients with recurrent bowel obstruction and a prior colorectal cancer history.

## Introduction

Appendiceal neoplasms are rare gastrointestinal tumors, identified in approximately 0.9-1.4% of appendectomy specimens, and encompass a heterogeneous spectrum of histologic subtypes, among which adenocarcinoma represents a clinically significant subset. Due to their rarity and nonspecific clinical manifestations, appendiceal adenocarcinomas are frequently diagnosed incidentally on histopathologic examination following surgery performed for unrelated indications [1-3]. The most common clinical presentation mimics acute appendicitis, while atypical presentations such as bowel obstruction are rare [2,4].

Small bowel obstruction (SBO) secondary to appendiceal adenocarcinoma has been described in the literature, often involving unusual mechanisms such as adhesive bands, mucoceles, or extrinsic compression rather than direct tumor invasion. Although early reviews identified only a small number of such cases, additional cases have since been reported, underscoring that this remains a rare but increasingly recognized clinical scenario [4,5].

The occurrence of multiple primary malignancies in a single patient, defined by the Warren and Gates criteria as histologically distinct tumors arising independently with exclusion of metastatic disease, is increasingly recognized, with reported prevalence ranging from approximately 2% to 17% depending on the definitions and detection methods used. These criteria require that tumors be verified by histology, arise as separate entities, with intervening non-neoplastic mucosa, and not be simply metastatic spread from the primary tumor [6,7]. Patients with colorectal cancer are at increased risk for second primary malignancies, and appendiceal neoplasms have been reported to occur in association with synchronous or metachronous colorectal tumors, highlighting the importance of thorough colonic evaluation [2,3].

We present a case of appendiceal adenocarcinoma presenting as recurrent SBO in a patient with a history of treated rectal adenocarcinoma, representing a metachronous primary malignancy. This uncommon manifestation adds to the limited existing literature and illustrates the diagnostic complexity of distinguishing a new primary from metastatic recurrence.

## Case presentation

A woman in her early 50s with a past medical history of type 2 diabetes mellitus, gastroesophageal reflux disease, and obesity presented to the emergency department with five days of nausea, vomiting, and diarrhea. She reported one day of absent output from her colostomy bag and sharp periumbilical abdominal pain.

Her oncologic history was significant for stage III (T4bN2a) rectal adenocarcinoma, diagnosed two years prior. The initial colonoscopy revealed a large, non-obstructing, fungating, ulcerated mass in the rectum. Staging MRI of the pelvis demonstrated a locally invasive low rectal tumor extending 8 cm craniocaudally, fully circumferential, with invasion through the internal sphincter, intersphincteric plane, and external sphincter into the perineum and posteroinferior vaginal wall, with five suspicious mesorectal lymph nodes (MR staging T4bN2a). CT of the chest, abdomen, and pelvis at initial staging showed no evidence of metastatic disease, and the appendix was noted to be normal. Following neoadjuvant chemoradiation, the restaging MRI demonstrated a partial response with significant tumor reduction and resolution of mesorectal lymphadenopathy. She subsequently underwent robotic-assisted laparoscopic abdominoperineal resection with total abdominal hysterectomy and bilateral salpingo-oophorectomy, achieving remission. Surveillance CT imaging at 6, 12, 18, and 24 months post-resection consistently demonstrated no evidence of recurrent or metastatic disease, and the appendix was explicitly described as normal on multiple examinations.

Surgical pathology following the abdominoperineal resection demonstrated ypT0N0 disease, indicating a complete pathologic response to neoadjuvant therapy, with no residual invasive tumor at the primary rectal site and no metastatic involvement of the regional lymph nodes. Approximately two years after the resection, and seven months before the current presentation, the patient experienced an initial episode of SBO. CT of the abdomen and pelvis at that time demonstrated dilated small bowel loops with an apparent transition point in the distal ileum, with the colostomy and left-sided colon decompressed. This episode resolved with conservative management.

At the current presentation, physical examination demonstrated abdominal distention and diffuse tenderness without peritoneal signs. CT of the abdomen and pelvis with intravenous contrast revealed recurrent features suspicious for SBO, including small-bowel dilatation and air-fluid levels more pronounced than on the prior examination (Figure 1A-1B). The terminal ileum was nondilated, with the transition point likely within the deep pelvis posteroinferiorly, where post-surgical stranding was chronic. Additional findings included a small volume of pelvic free fluid and new mild right-sided hydroureteronephrosis without a radiodense stone, with the distal right ureter likely at least partially obstructed at the same level. Tissue thickening and distortion in the presacral space were unchanged and presumed post-surgical and post-radiation related. No lymphadenopathy or hepatic masses were identified.

**Figure 1:**
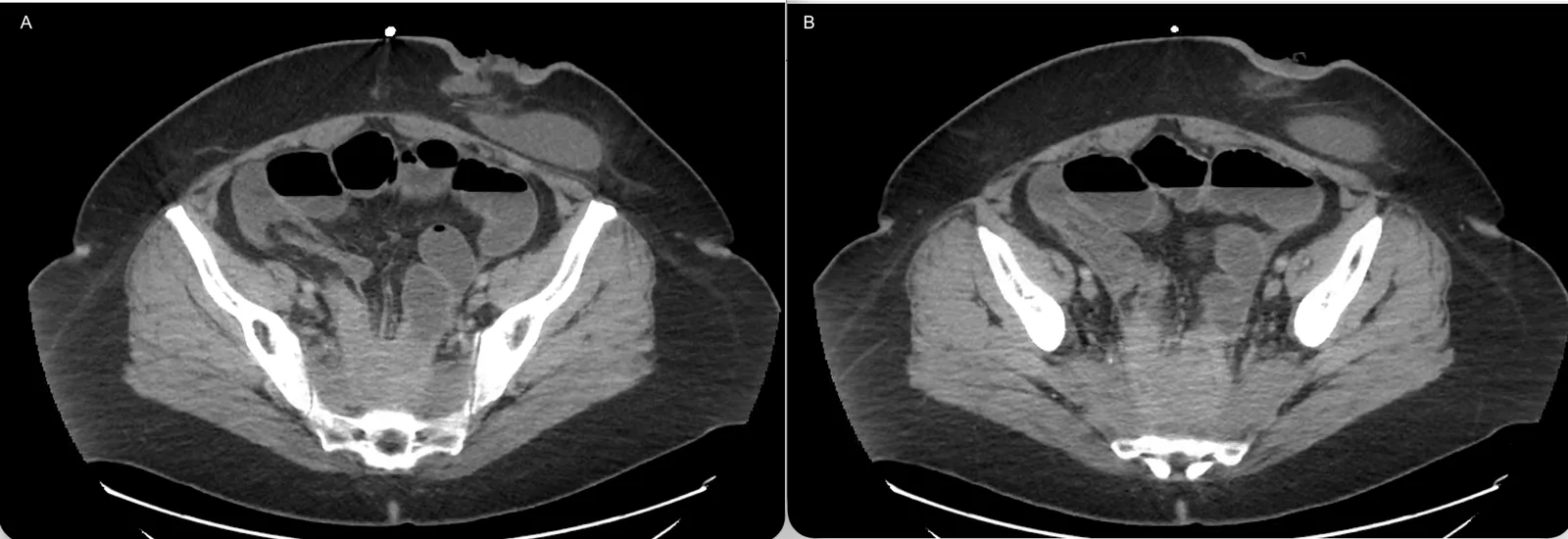
Axial contrast-enhanced CT images of the abdomen and pelvis demonstrating findings consistent with SBO. (A) Dilated, fluid- and gas-filled small bowel loops with fecalized intraluminal contents, consistent with the small bowel feces sign. (B) More caudal image demonstrating decompressed bowel near the suspected transition zone, supporting a mechanical SBO CT: computed tomography, SBO: small bowel obstruction

Initial management included nasogastric decompression, which failed to resolve the obstruction. The patient subsequently underwent operative intervention, and an ileocecectomy was performed due to the terminal ileum and cecum being densely adherent within the previously irradiated pelvis. Gross examination of the resected specimen revealed a segment of cecum and terminal ileum with extensive serosal adhesions and a strictured area with serosal disruption suggestive of perforation. The appendix was extensively adherent to the surrounding tissues without a discrete gross mass; the distal tip was disrupted and hemorrhagic at the area of adhesion, also suggestive of perforation.

Histopathologic examination demonstrated a moderately differentiated adenocarcinoma arising from the distal appendix, measuring 2.0 × 1.2 × 1.0 cm. The tumor exhibited perforation with direct invasion into the subserosa of the adjacent small bowel. Lymphovascular invasion was identified. Surgical margins were negative, and all eight regional lymph nodes were negative for metastatic carcinoma, yielding a pathologic stage of pT4bN0. Notably, high-grade dysplasia was identified in the adjacent appendiceal mucosa, with the tumor effacing the appendiceal architecture, consistent with a primary appendiceal neoplasm (Figures 2-3). No therapy-related effects were identified.

**Figure 2:**
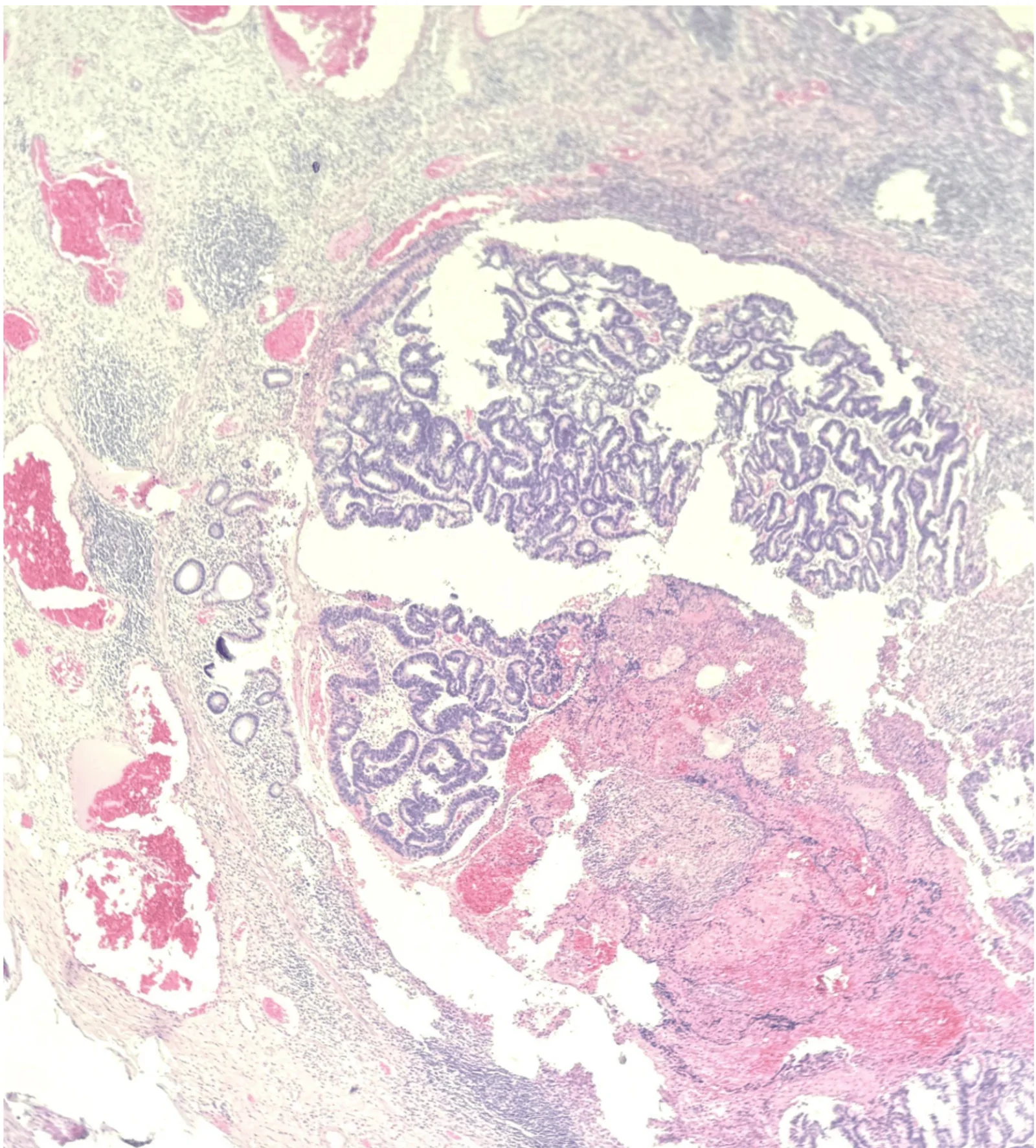
Low-power histologic view of appendiceal adenocarcinoma filling the lumen (H&E stain, 4× magnification) H&E: hematoxylin and eosin

**Figure 3:**
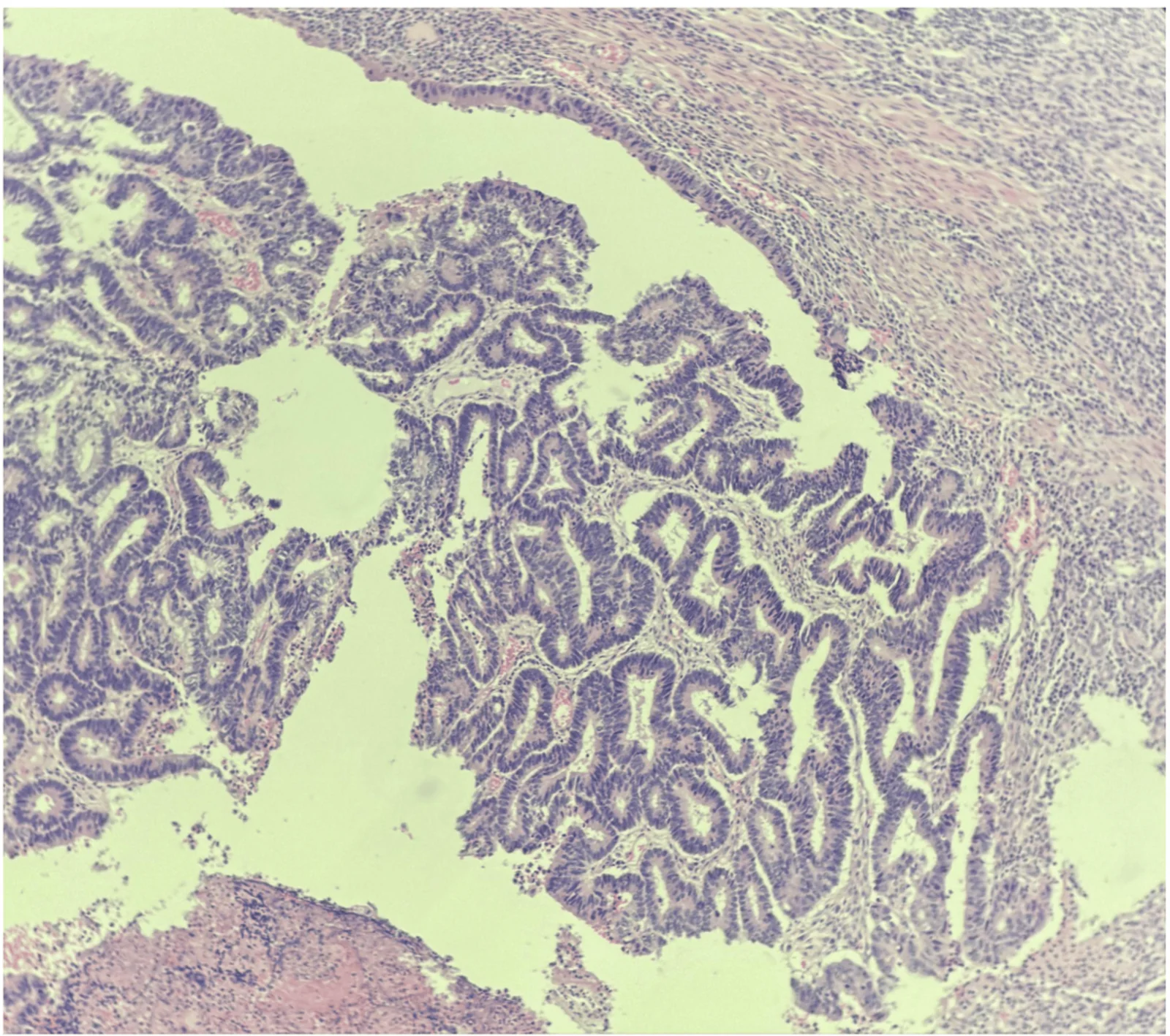
High-grade dysplasia in appendiceal epithelium (H&E stain, 10× magnification) H&E: hematoxylin and eosin

Immunohistochemical staining demonstrated diffuse CDX2 positivity (Figure 4), focal CK20 positivity, and CK7 negativity, consistent with an intestinal/colonic-type adenocarcinoma. This immunophenotypic profile, combined with the presence of in situ dysplastic changes (Figure 3), the anatomic confinement of the tumor to the appendiceal architecture (Figure 5), and the absence of treatment-related histologic effects, confirmed the diagnosis of a primary appendiceal adenocarcinoma rather than a metastatic recurrence of the previously treated rectal carcinoma.

**Figure 4:**
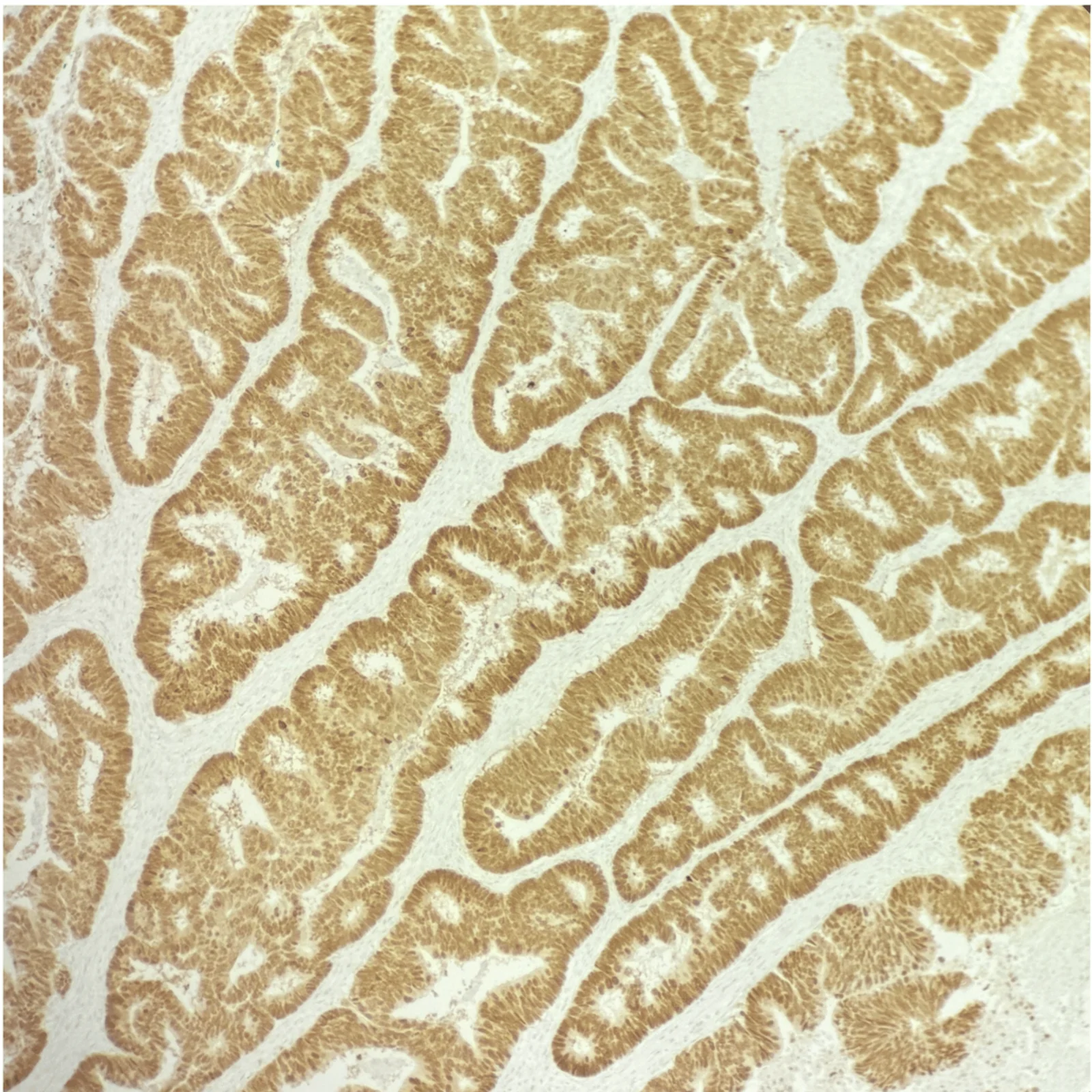
CDX2 immunohistochemical staining demonstrating intestinal differentiation (CDX2 immunostain, 10× magnification)

**Figure 5:**
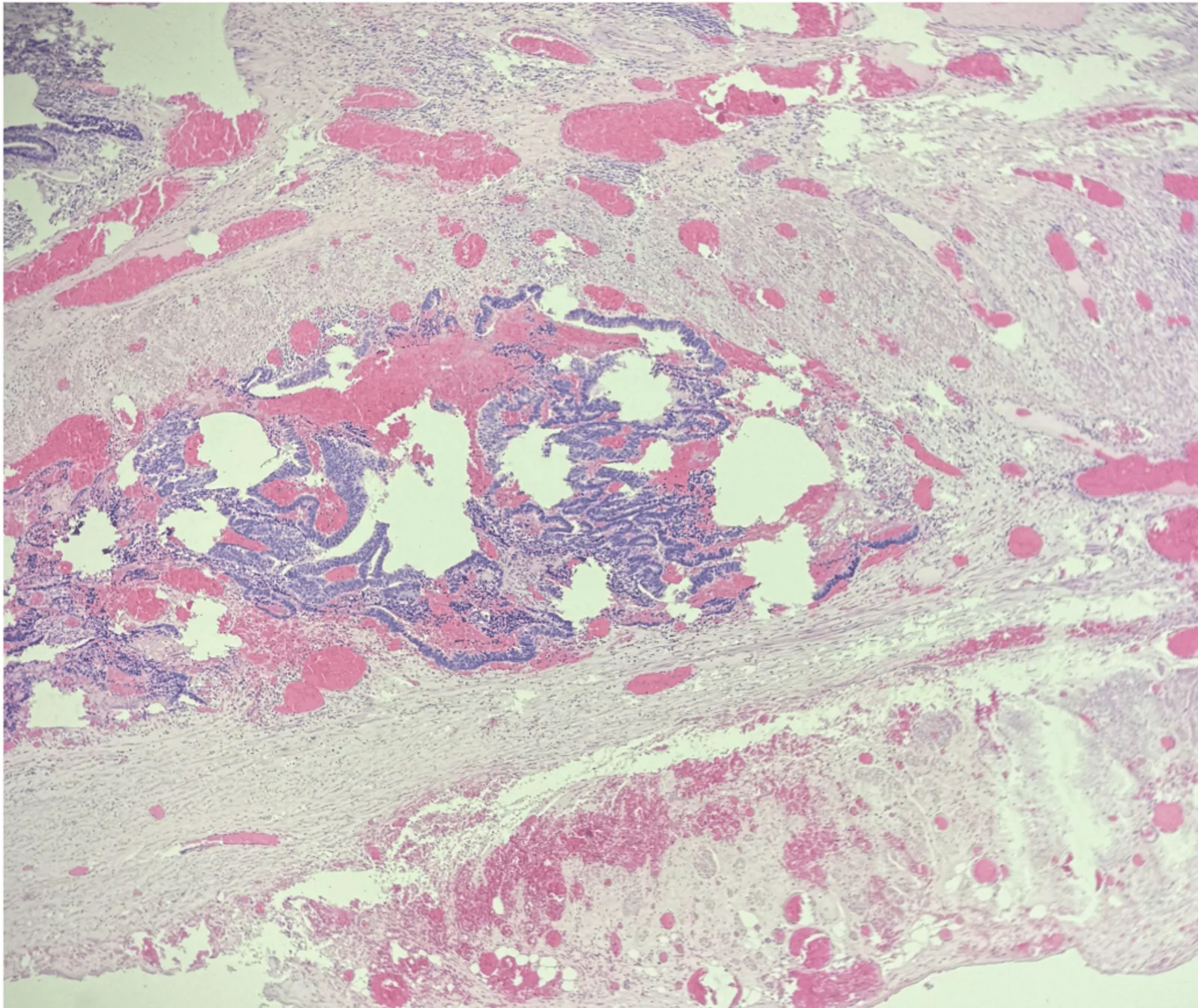
Tumor invading muscularis propria of appendix (H&E stain, 4× magnification) H&E: hematoxylin and eosin

Postoperative surveillance CT of the chest, abdomen, and pelvis performed approximately three months after ileocecectomy demonstrated interval placement of a right-sided nephroureteral stent with decompression of the right kidney, an ileocolic anastomosis in the right lower quadrant, and no evidence of thoracic, hepatic, or nodal metastatic disease (Figure 6A-6B). The nephroureteral stent was placed at the time of surgery to address hydronephrosis and to identify and protect the ureter. No bowel obstruction was identified.

**Figure 6:**
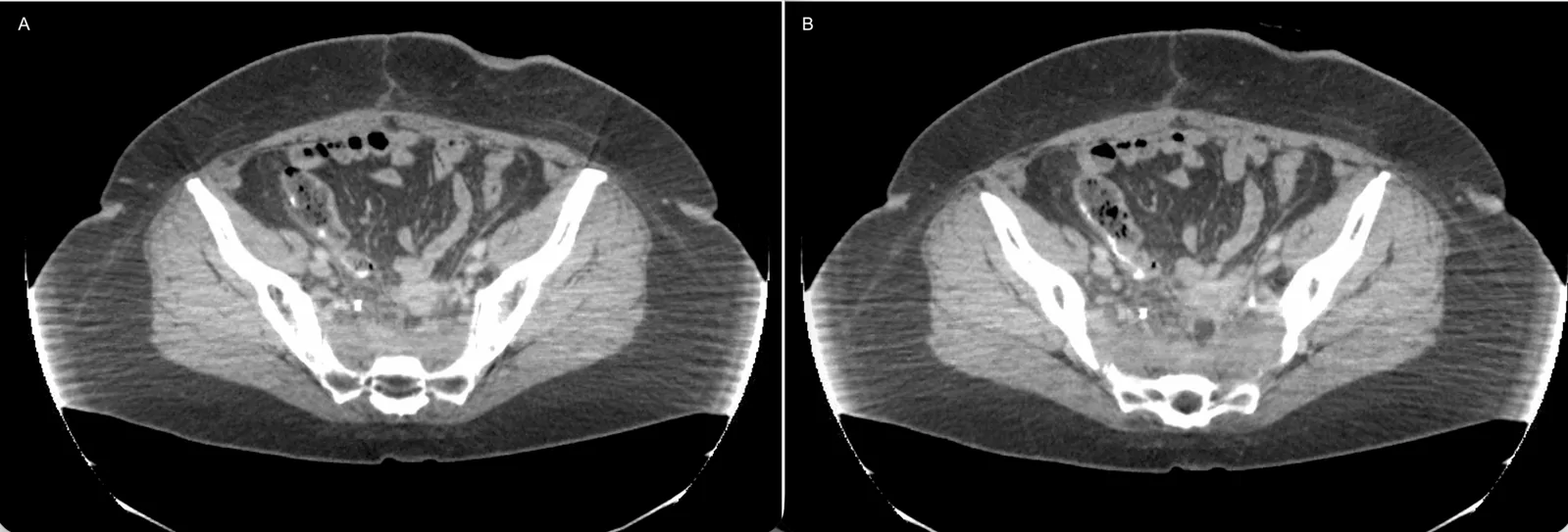
Postoperative surveillance CT of the chest, abdomen, and pelvis performed approximately three months after ileocecectomy CT: computed tomography

## Discussion

Primary appendiceal adenocarcinoma is a rare malignancy, accounting for approximately 1% of gastrointestinal cancers [1-3]. The majority of cases are diagnosed incidentally following surgery performed for suspected appendicitis or other abdominal pathology. Although appendicitis-like symptoms represent the most common presentation, mechanical SBO secondary to appendiceal malignancy is exceedingly uncommon. A review of the literature suggests that fewer than a couple of dozen cases of appendiceal adenocarcinoma presenting with SBO have been described [4,5].

In previously reported cases, obstruction often results from unusual mechanisms such as appendiceal mucoceles, inflammatory adhesive bands, or extrinsic compression rather than direct transmural invasion [4]. The present case is notable for recurrent SBO associated with extensive serosal adhesions likely attributable in part to prior surgical procedures for rectal carcinoma and tumor perforation. These findings contributed to obstruction at the terminal ileum and ultimately necessitated ileocecectomy, with the underlying malignancy identified only on subsequent histopathologic examination.

Analyzing the radiographic timeline, the appendix was explicitly described as normal on multiple surveillance CT examinations over a two-year period following treatment for rectal carcinoma, including as recently as six months before the obstructive presentation. This demonstrates a recognized limitation of cross-sectional imaging in detecting early or small appendiceal neoplasms, particularly in the absence of associated mucocele formation or significant appendiceal dilation [3]. In our case, the absence of radiographic abnormalities despite the subsequent discovery of a 2.0 cm tumor with transmural invasion and perforation highlights the occult nature of appendiceal malignancies and the limitations of imaging alone for their detection.

A critical diagnostic consideration in this case was the distinction between a new primary malignancy and metastatic recurrence from the patient's prior rectal adenocarcinoma. According to the Warren and Gates criteria, multiple primary malignancies are defined by three conditions: each tumor must be histologically confirmed as malignant, each must be geographically distinct, and the possibility that one tumor represents a metastasis of the other must be excluded [7]. These criteria were met in this patient, as both tumors were histopathologically confirmed, they arose in anatomically distinct areas, and the appendiceal lesion demonstrated architecture supporting its classification as an independent primary tumor rather than a metastatic recurrence. In the present case, several features supported the diagnosis of a metachronous primary appendiceal adenocarcinoma: the presence of high-grade dysplasia in the adjacent appendiceal mucosa, demonstrating a transition from dysplasia to invasive carcinoma; the tumor's location effacing the appendiceal architecture; and the absence of therapy-related histologic effects. The immunohistochemical profile (CDX2+, CK20 focal+, CK7−) was consistent with a lower gastrointestinal primary but could not alone distinguish between appendiceal and rectal origins; however, the histopathologic identification of in situ precursor changes strongly supported a primary appendiceal neoplasm.

The association between appendiceal tumors and synchronous or metachronous colorectal neoplasms is well documented [2,3]. Previous studies have reported an increased incidence of additional primary malignancies in patients with appendiceal adenocarcinoma, with the majority located in the gastrointestinal tract, and primary malignant appendiceal tumors have been associated with synchronous or metachronous colorectal neoplasms [2,3,8]. These data demonstrate the importance of comprehensive colorectal evaluation in patients with appendiceal neoplasms and, conversely, vigilance for appendiceal pathology in colorectal cancer survivors [2,3].

Regarding surgical management, right hemicolectomy with adequate lymphadenectomy is recommended for appendiceal adenocarcinoma to allow for comprehensive lymph node staging [2,9]. However, limited resection such as appendectomy or ileocecectomy with negative margins and adequate lymph node sampling may be sufficient in select cases. AlMasri et al. demonstrated that appendectomy was oncologically equivalent to right hemicolectomy for well-differentiated T1 appendiceal adenocarcinoma [9]. In the present case, the ileocecectomy achieved negative surgical margins with eight negative lymph nodes. However, the advanced stage (pT4b) with perforation and lymphovascular invasion carries a higher risk of peritoneal dissemination and warrants close surveillance. Given the patient’s clinical and surgical history, the surgical team elected not to perform another major surgical procedure to obtain additional lymph nodes. This case highlights the importance of maintaining a broad differential diagnosis in patients with recurrent SBO, particularly those with a history of prior malignancy. It emphasizes the critical role of thorough pathologic evaluation in identifying occult neoplasms.

This single-case report has several limitations. The findings are descriptive and do not establish causality or the frequency with which appendiceal adenocarcinoma presents as recurrent SBO. Additionally, this case highlights the limitations of surveillance CT in detecting occult appendiceal malignancy, and the observations should not be interpreted as evidence against routine surveillance imaging. To better define the optimal diagnostic approach and management of these uncommon presentations, further accumulation of similar cases is needed.

## Conclusions

This case illustrates an uncommon presentation of appendiceal adenocarcinoma discovered incidentally following ileocecectomy for recurrent SBO, representing a metachronous primary malignancy in a patient with a history of stage III rectal adenocarcinoma. Serial surveillance imaging over two years had demonstrated a radiographically normal appendix, underscoring the occult nature of this malignancy and the limitations of cross-sectional imaging. Clinicians should consider rare malignant etiologies in patients with recurrent obstructive symptoms, particularly those with a history of colorectal cancer. As demonstrated in this case, establishing the correct diagnosis requires integrating the patient’s clinical history with histopathologic evaluation and immunohistochemical findings, rather than relying solely on surveillance imaging. Comprehensive histopathologic assessment, including evaluation of in situ precursor lesions and immunohistochemical profiling, remains essential for distinguishing primary from metastatic disease and guiding optimal management.
